# Practice variation amongst preventive child healthcare professionals in the prevention of child maltreatment in the Netherlands: Qualitative and quantitative data

**DOI:** 10.1016/j.dib.2017.09.061

**Published:** 2017-10-02

**Authors:** Simeon J.A. Visscher, Henk F. van Stel

**Affiliations:** Department of Healthcare Innovation and Evaluation, Julius Center for Health Sciences and Primary Care, University Medical Center Utrecht, Netherlands

## Abstract

This article provides both qualitative and quantitative data on practice variation amongst preventive child healthcare professionals in the prevention of child maltreatment in the Netherlands. Qualitative data consist of topics identified during interviews with 11 experts (with quotes), resulting in an online survey. The quantitative data are survey responses from 1104 doctors and nurses working in 29 preventive child healthcare organizations. Additionally, the interview topic list, the qualitative data analysis methodology, the survey (in English and Dutch) and anonymized raw survey data (http://hdl.handle.net/10411/5LJOGH) are provided as well. This data-in-brief article accompanies the paper “Variation in prevention of child maltreatment by Dutch child healthcare professionals” by Simeon Visscher and Henk van Stel [1].

**Specifications Table**

**Table:** 

Subject area	*Medicine*
More specific subject area	*Prevention of child maltreatment by preventive child healthcare professionals (physicians and nurses)*
Type of data	*Tables, text file, graph*
How data was acquired	*Semi-structured interviews, followed by an online questionnaire*
Data format	*Raw*
Experimental factors	*N/A*
Experimental features	*A survey was derived from the semi-structured interviews. Cross-sectional survey in 30 organisations for preventive child healthcare. Percentages for response categories were computed.*
Data source location	*The Netherlands*
Data accessibility	*Data is included in this article, full (anonymized) survey data available at*https://dataverse.nl/dataset.xhtml?persistentId=hdl:10411/5LJOGH
Related research article	*Variation in prevention of child maltreatment by Dutch child healthcare professionals (Child Abuse & Neglect 70 (2017) 264–273)*

**Value of the data**

• Lists 38 topics that interviewed experts believe are vital in the prevention of child maltreatment, yet are not commonplace at the moment
• Provides response distributions for each questionnaire item separately
• Can be used to prioritize specific quality improvement efforts
• May inspire other practice variation studies, because the data provides a frame of reference and extra insight into our methods enhances reproducibility of each step during the process
• Provides the English and Dutch versions of the survey on prevention of child maltreatment by preventive child healthcare professionals

## 1 Data

First, we present the interview methodology, including the selection and the characteristics of the experts we interviewed. Second, we describe the qualitative methods used to analyse the interviews. Third, topics emerging from the qualitative analysis are listed, which the experts considered important in the prevention of child maltreatment, yet, of which they expected a large amount of practice variation would be present. Fourth, we present the questionnaire we used to verify this hypothesized practice variation. For each multiple choice question the response distribution is displayed. For some questions additional data are provided.

### 1.1 Interview methodology

Individual interviews were conducted, for which the subjects were recruited using purposive sampling [2]. The objective was to select individuals who should be qualified to define optimal care in this area. The interview was based on a topic guide, which was augmented with new topics after each interview. The initial topic guide was based on the guideline and the current literature [3], [4]. After informing the participant of matters such as anonymity and study objective, the interview started with a broad open-ended question [5]. As the subject responded, neutral probing questions were asked to identify specific topics, and to determine the relevance of each topic for inclusion in the questionnaire. Subsequently, topics were introduced, which had not yet been mentioned spontaneously by the interview subject. Once the topic guide became too lengthy to cover each topic in every interview, topics that were satisfactorily discussed in the foregoing interviews were omitted. New interviews were conducted until data saturation occurred (i.e. no new topics or viewpoints came up). Several techniques were used to improve the quality and validity of the data, including audio recording and member checking of the transcripts [5], [6] (Table 1, Table 2, Table 3, Table 4, Table 5).

**Table 1:** Recruitment of subjects.

*Domain*
Professional association of preventive child healthcare physicians
Educational committee
Professional association of preventive child healthcare nurses
Scientific committee
Netherlands School of Public Health, department child health care
Educational committee
TNO, department child healthcare (The Hague)
Educational committee
Committee for revision of the child healthcare guideline on prevention of child maltreatment
National expertise centre child maltreatment (LECK)
Two authors of medical books on child maltreatment
Referrals from members of the above-mentioned groups
*Invitation*
By telephone (if the phone number could not be found per email)
Study objective and interview objective were mentioned
Location was determined by participant
Time duration 45–60 minutes
No financial compensation

**Table 2:** Interview design.

*Schedule*
Introduction (see “Techniques to improve quality of the interview data”)
Full-breadth open-ended question [5], enhanced by:
Neutral probing questions
Probing questions to ensure coverage of all aspects
Skills, knowledge, methods and improvement opportunities
Importance, as well as expected amount of practice variation
Clarifying questions and summaries
Introduction of items from the topic guide that were not mentioned spontaneously
Question about coverage of the interview (i.e. “Where all major topics discussed?”)
After each interview: topic guide update (based on notes from during the interview)
*Initial topic guide*
Skills
Communication
Assertiveness
Building up a relationship with the parents
Discussing sensitive topics
Open-mindedness
Tactful language
Transcultural communication
Other
Knowledge
Detection of maltreatment
Signs and risk factors unrelated to physical examination
Physical examination
Responding to maltreatment
Contents of the child healthcare guideline on secondary prevention of child maltreatment
Legislation
Practical knowledge/Other
Acquaintance with professionals from other agencies
Child protection services
Primary school
General practice
Paediatrics
Other
Practices
Adherence to the child healthcare guideline for secondary prevention of child maltreatment
Adherence to national reporting laws
Self-improvement (e.g. studying, reflection)
Activities to promote acquaintance with other agencies

**Table 3:** Interview technique.

*Establishing a safe environment*[5]
Before the interview started
Location determined by participant
Personal introduction
Confidentiality guaranteed
Permission asked to audiotape the interview
The possibility to verify the transcript was mentioned
The participant was asked if s/he had any questions
Rapport developing techniques
Broad, open-ended, non-threatening opening question
Repeating the interviewee's words
Probing also on less relevant narratives
*Preventing miscommunications*
Definitions provided before beginning of the interview
Clarifying questions and summaries to verify understanding
Audio recording (digital voice recorder, Sony ICD-AX412 F)
Literal transcription and member check of all transcriptions
*Minimizing researcher's bias*
Neutral explanation of the study objective (i.e. little information on study hypotheses)
Non-directive probing questions
Topics that were not discussed after the open-ended question were introduced at the end of the interview
*Interviewer's experience*
Some experience in interviewing in general (ca. 20 interviews in the year prior to the study)
No experience in scientific interviewing

**Table 4:** Coding methods.

*Coding**[7]*
The researcher was already familiar with the data
Design of the interview schedule and its topic guide
Administration of the interview
Transcription of the interview
Node list
The topic guide served as an initial list of nodes.
If a passage was eligible for multiple nodes, each node was first precisely defined. If the problem persisted, the passage was multicoded.
New codes were created as additional concepts were identified. Existing nodes related to the new nodes were fully recoded.

**Table 5:** Scoring methods.

*Scoring*
Principles
Goal of the scoring system is to prioritize topics relative to each other, for selecting items for the questionnaire
Focus on relative credibility, instead of absolute credibility
Overall impression of the researcher is conclusive
Primary focus on the literal meaning of the text and triangulation
Secondary focus on the context of the passage
Tertiary focus: quantitative analyses performed in NVivo (word count, passage count, interview count, interview coverage (i.e. word count divided by total interview word count) and overlap with other nodes)
Triangulation
Opinion of one participant: devaluation of literal meaning of the text
Opinion of two or more participants: inclination towards literal meaning of the text
Opinion of three or more participants: acceptance of literal meaning of the text
Triangulation can be overruled by compelling arguments, such as:
Scientific evidence
Legitimate examples and a reasonable explanation of underlying mechanisms
Participant is especially accomplished in the particular area
The participant finds it extremely important, at least supported by a high interview coverage
Spontaneously shared information has more value than answers to specific questions
Importance
Definition: How important this topic is for the efficacy of primary or secondary prevention of child maltreatment.
++ Very important
+ Important
± It helps
- Not important, but it probably helps
-- Not important, and it probably does not help either
Expected amount of variation
Definition: The amount of improvement that could probably be made through change of policy.* This measure is a composite score of how *many* professionals could improve in this area, and how *much* they could improve in this area.
* = The influence of policy was estimated by the researcher
++ most professionals could improve a lot
+ it is not uncommon that professionals could improve a lot OR most could improve moderately
± it is not uncommon that professionals could improve moderately OR most could improve a little
- it is not uncommon that professionals could improve a little in this area
-- only exceptional cases could improve in this area OR the influence of policy is small
Expected amount of response bias (conjecture of researcher)
Definition: The amount of bias the researcher (subjectively) expects, considering the fact that only a few brief questions can be used, and taking into account social desirability, probability of overestimation, subjectivity and complexity.
++ no notable consequences are expected
+ a meaningful estimation of the effect size is expected to be possible
± a meaningful estimation of the effect direction is expected to be possible
- a meaningful estimation of the effect direction may be possible, dependant on the average tendency towards response bias
-- this item should not be measured by a short question, because interpretation of the results will not be possible.
Overall relevance score
++: Very relevant - (all items ≥ ±) AND (≥ 2 items = ++)
+: Relevant - (all items ≥ ±) AND (≥ 1 item ≥ +) AND (≤ 1 item = ++)
±: Somewhat relevant (all items = ±) OR ((2 items = ++) AND (1 item = -))
-: Barely relevant (≥ 1 item = -) AND (< 2 items = ++)
--: Not relevant (≥ 1 item = --)

### 1.2 Interview participants

Eleven experts were interviewed (age 30–68 yr), including 7 physicians, 3 nurses and 1 health scientist. Several participants were highly experienced because they either worked at child protective services (n=1), taught prevention of child maltreatment (n=3), or occupied the position of “designated expert” at their own child healthcare organization (n=4). The latter involves being available for consultation by colleagues, which is standard procedure according to the national guidelines, every time a child healthcare professional suspects maltreatment. Other participants (co)authored a book on child maltreatment (n=2), or contributed to the current guideline (n=4) and/or its upcoming revision (n=4). Professionals active in both age categories, 0–4 and 4–19 yr participated.

### 1.3 Qualitative analysis

The qualitative data-analysis consisted of organizing and interpreting the data. The first part was done by thematic analysis: assigning codes to chunks of transcribed text relating to a particular topic [7] (using NVivo 10 [8]). Once all substantive text was coded, an overview was generated for each code, containing the passages related to the topic. Subsequently, these overviews were interpreted and an overall summary was made. Whilst summarizing, a five-point scale was used to describe the importance of a topic, and its expected amount of variation. Based on the summary, an overall score was calculated, which represented the overall relevance for inclusion in the questionnaire. A detailed description about the interpretation process can be found below. Finally, the topics were classified into categories.

Topics

**Table:** 

*Domain 1: Communication*	
**Topic**	**Description**
Openness	Openly communicating suspicions towards the parent, as opposed to secretively collect evidence against him/her. Child maltreatment is almost always the cause of pedagogical incompetence, rather than malice. The first step should almost always be to communicate your observations with the parents.
	*“You shouldn’t have a hidden agenda.”*
	*“You have to dare to openly share your worries.”*
Open-mindedness	Communicating to a client that you will not judge them, so that they can tell you anything.
	*“This part also has to do with norms and values. (…) for instance, a mother who kept her child on a leash outside, because her child would otherwise run away. [You mean like people normally do with their dogs?] Yes. And there were many other things as well. But yes, I found that abnormal. And that is not per se directly child maltreatment, but it was a whole aspect, she also… (…) I think that's close to child maltreatment. Or maybe it is child maltreatment, systematically, because the child also had all kinds of behavioural problems. While she thought: yes but we’re doing so well. I don’t see the problem and uhh… I’m sharing this with you now, but you are judging me for it. Well, from now on I won’t share anything anymore. (…) So you need to be able to empathize with why the parents do things. Projecting yourself into their reference frame.”*
	*“Especially things like sexual abuse, are veeery sensitive topics. It is veeery hard to make that discussable, to then just think like, what could this be? Because, and to then, not have a verdict on that, but to openly discuss it with the parent…”*
Conversational skills	All kinds of conversational skills and techniques are needed, such as rapport developing techniques (e.g. always giving a compliment about the child, showing interest in the other, active listening, communicating empathy), being able to uncover an underlying question behind a question or an underlying problem behind a problem, summarizing, taking heed of the client's expectations, using pauses and silences in order to let the other person speak, structuring consultations (e.g. getting back on topic if one has landed on an irrelevant side track) and motivational interviewing.
	*“The communication that you should have as a child healthcare physician, the competences therein, that is really of the highest level. [Does it? Does it have to be the highest level?] Yes, that is so tremendously important.”*
Adjustment to the person in front of you	Adjusting to the person in front of you: the client's culture (e.g. from another country, but also people with a low social economic status can be considered a (sub)culture), his/her intelligence, language level, home situation, upbringing of the parents themselves, etc.
	*“We are highly educated, remember, we know a lot, and we assume too much that people already know things.”*
	*“Sometimes people say things of which I think, I really don’t know what they’re talking about, and then I have to ask them as well.”*
	*“You have to be able to explain it to them, I mean, you have to understand their line of reasoning in order to explain what you… So that it is understandable for them why you’re worried.”*
Assertive communication skills	Motivating parents to accept help, if necessary by applying pressure, standing up for a child: *“Parents may say: ‘I’m not obligated to attend.’ Then you say: ‘No, but according to the Convention on the Rights of the Child, your child has the right to preventive health care.’”*
	*“We see a relatively large group who finds it difficult. They will not take a stance if the parent does not want to accept the things that are necessary to improve the situation, who may threaten, who may file complaints et cetera. (…) They are easily cornered.”*
	Being able to introduce a sensitive topic too, requires strong assertive communication skills. The same goes for standing up to your manager, if it be necessary: *“If you have an anxious manager who does not dare to say to the city counsel member: until here and no further, (…) [What influence does a child health care physician have on such a situation?] He can say to his manager like, listen, this is my specialty, this is my work and I have done this in such a way, (…) you only have to be able to explain it.”*
Flexibility	Professionals have to be flexible during a consultation: *“You have to be very flexible, quickly adapting to their current questions. (…) You have to dare to let go of the structure of your examination. Normally you examine the eyes, ears, length, weight, etc., but this kind of conversations often goes chaotically. Because people at that moment have nee… you know, if a mother of a child has a need at that moment to say something, you have to listen to that. You shouldn’t say then, yes wait a moment, I am typing. Because they can’t do that. So you have to be flexible.”*
	*“It's about being flexible in that kind of stuff. [Yes. I’ve got the feeling you are also trying to say where there's a will, there's a way.] Yes, especially in this area.”*
Communicating with children	Being skilled at communicating with children in specific, and being able to interpret the parent-child-interaction.
	*“If a child says that daddy was very angry, and you say, well, did smoke come out of daddy's ears, or that kind of stuff, and the child goes on with it, and says yes, there came so much smoke out of his ears and then he exploded! And a parent is sitting next to the child and thinks well let him babble… (…) Parents can of course brilliantly tell how well they are doing, whereas children can also say they’re okay, but I don’t like this, or I don’t like that, and… (…) children are of course part of the story.”*
	*“Being able to pick up signals about does a child feel safe or do they feel unsafe? A child who has never seen me, walks into my consultation room and, just like that climbs on my lap, I’m worried about such a child. That kind of signals.”*
Diplomacy	Being skilled at using diplomatic/tactful language. Prioritizing in situation with multiple problems that need to be solved. Being able to negotiate with parents. Being able to bring a message across, without ruining the relationship.
	*“If say to a Moroccan father like, in Holland it's forbidden to beat a child, in common, so, the teacher isn’t allowed to do it, I don’t offend him, but then he knows it. And he will act like he already knew that, but in the meantime he thinks hmm…”*
***Domain: Medical expertise***	
**Topic**	**Description**
Knowing all risk factors and symptoms	Knowing all risk factors and symptoms of child maltreatment. After openly communicating suspicions with parents, this was regarded the most important topic of all.
	*“So you have to know the signals and all, the hundreds of signals and symptoms very well, of child abuse, maltreatment and sexual abuse.”*
	*“Sensitivity, but also knowledge. You also have to know what all the possibilities are, when it comes to risk factors and signals.”*
	*“Then I wonder is he appropriately dressed for the season? You know, that kind of stuff.”*
	*“When it comes to babies, half a year of age, you can always easily see if the attachment is secure. If I examine that child, and I am standing above that child, then I am a threat. And what are you then supposed to see: a child of half a year old looks at the mother. And s/he reads the face of the mother, the mother smiles, then it is okay. That is a sign for me that the child is safely attached to his mother. Yeah, I’ve got that from a book. But once you’ve seen it, you see it each time again.”*
	*“There are a number of books and articles that you have to, simply facts about how often things take place, well you name it, what it can look like, et cetera, the whole start-up phase, what do you do, it's all knowledge that you have to possess.”*
Knowledge of guideline on prevention of child maltreatment	Knowledge of the child healthcare guideline secondary prevention of child maltreatment. Quote referring to the guideline: *“You just have to know it, you have to possess that knowledge”*
	*“[So if would do e-learning modules, and…]…the guideline, then you are well on your way, yes.”*
	*“I think our profession [child healthcare nurses], well, I don’t want to be condescending, but still, that they hardly read any literature, or perhaps just a little.”*
	Some experts asserted that the knowledge has to reach beyond the guideline: *“Yeah… Don’t get me wrong, [the guideline] is good, and it is being updated at the moment. It contains quite a lot, but eventually it is insufficient. In the end, you need to read other things next to it, you have to study other material as well, you need to grab a book.”*
Considering the context of the client	Asking about the current situation/context of the child. This includes a so called parent check: asking about alcohol or drug addiction of the parents, financial problems or stress, stress at work, relationship difficulties, psychopathology, maltreatment history of the parents and spousal abuse. Not only exploring the physical health of the child, but all aspects.
	*“And you have to, the parent check I call that, as preventive child healthcare we have the parent check. In psychiatry they have to do a child check, if you meet a schizophrenic father, mother or a depressed mother, you have to ask them, like, do you have children, and how are your children doing. And we are of course, because we’re here for the children, have to know how the parents are doing. (…) So if dad is a schizophrenic, is he admitted? Does he take his medication, or is he unpredictable? Or is he paranoid? And does he bring that over to his children?”*
Preventively treating or referring children with mild behavioural problems	Children with behavioural problems should easily be referred to a psychologist or child rearing specialist (Dutch: *“(ortho)pedagoog”*)
	*“When I sit here, and I’m doing consultation hours, and I’ve got my drawer here, and little John aged two constantly walks to my table and opens my drawer. And a mother is sitting there, she says: [speaking with a foolish tone] Ahhh, he is so undertaking, isn’t he? [Laughing] Yes, it happens! How would we be able to… so I say, I don’t find that undertaking, I find that, I say, well, maybe you could keep him with you. (…) Or children who constantly turn light switches on and off, you know. So. Then I send them to a child rearing agency. (…)We choke in the number of child rearing specialists, all unemployed. (…) Sometimes they feel offended (…) If I say like, I believe you should go to a child rearing specialist, I think that's a good idea. We won’t be able to solve this with just the two of us. But it is prevention of child maltreatment. (…) Then you can also prevent child maltreatment, then you are preventing it from happening.”*
Precisely knowing relevant legislation	Knowing all relevant laws and protocols reduces fear of breaching medical confidence and/or suffering from legal consequences. It also provides the professional grounds to negotiate with parents who are refusing help.
	*“I also say, it's in the law. You know, and very often I refer to the Convention on the Rights of the Child. Many people don’t know (…) what articles are in it.”*
Interpreting injuries found during examination	Theoretical knowledge of injuries caused by child maltreatment, being able to bring that knowledge into practice.
Sensitivity towards signs	Being sensitive towards each and every sign of child maltreatment.
Knowledge of paediatrics	Knowledge about conditions that are caused or influenced by child maltreatment, knowledge about conditions that can resemble child maltreatment, knowledge about normal development (in order to know abnormal development as well).
***Domain: Collaboration***	
**Topic**	**Description**
Acquaintance with other disciplines working in the same neighbourhood	Ensuring that all people who work with children in the same area, have a low threshold of contacting you in case of suspecting child maltreatment. These include, dependent on the feasibility:
	• all general practitioners in the neighbourhood • all paramedics *(speech therapists, physiotherapists, dieticians, etc.)* • all child rearing experts *(Dutch: “(ortho)pedagoog”)* and child psychologists • all social workers • all youth care workers • the relevant city counsel member(s) • all or at least one of the paediatricians in the nearest hospital • physicians and nurses of the emergency department in the nearest hospital, or a designated contact person • the child maltreatment attention officer of the nearest hospital • one or more child protective services employees • one or more police officers in the neighbourhood • for professionals working with clients aged 0–4 yr: a. midwives b. maternity nurses c. preschool personnel d. special education services for pre-schoolers with disabilities • for professionals working with clients aged 4–19 yr a. school social workers b. school guidance counsellors c. teachers d. school executives
	*“You should have talks with everyone, well we have mentioned I don’t know how many organizations who could all have information. And, you are not always supported in that by an organization, so this is such an essential thing, that you have to find out.”*
	*“You really have to, you should know everyone on a neighbourhood-level by name and phone number. (…) Ideally in person, but sometimes that's just not feasible. For instance in Amsterdam.”*
Organizing events	Organizing events for parents and/or teachers in which the professional gives advice on child rearing or prevention of child maltreatment.
	*“[At what places do you have to organize such an event?] On neighbourhood level. [On neighbourhood level, you mean…] At a school… I used to attend schools. I had this school (…) and then I organized a coffee morning about hitting, healthy food, every subject is spoken about, is treated, and then they know who you are, what you stand for, and then they do come to you when there are problems.”*
	A professional can also organize events for his own colleagues, perhaps in collaboration with child protective services (see also: frequent education).
***Domain: Involvement***	
**Topic**	**Description**
Overall involvement related to child maltreatment	Being involved in the prevention of child maltreatment on micro-, meso- and macro level: on a case level, but also involvement on a neighbourhood level (e.g. *“You know easy, that it's is not, but that you say like, you know it occurred to me that we see a lot of teenage mothers in this neighbourhood. How can we make sure that these teenage mothers do well?”*), on the municipality level (e.g. influencing the policy of the municipality) and on larger levels (e.g. teaching about signals of child maltreatment at a regional educational institute for teachers, or being politically active).
	*“There are people who really, how shall I put it, well who read about it and that's it, but you have to integrate it in your, in your whole professional profile, it has to be intermingled. It shouldn’t be yet another subject. (…) It consists of being* able *to,* daring *and* willing *to see child maltreatment.”*
Consultation of Child Protective Services	Always contacting CPS when child maltreatment is suspected. Many professionals think CPS is only meant for filing a report of child maltreatment, but this is not the case. They usually offer invaluable guidance on how to communicate with parents and which subsequent steps should be taken. In the Netherlands it is mandatory that professionals who work with children, get in touch with CPS when they have suspicions of child maltreatment. Since the interviewed experts estimate that 10–20% of the professional's workweek should primarily be focused on preventive activities related to child maltreatment, the number of consultations with CPS should be substantial.
Consultation of internal child maltreatment expert	In the Netherlands every preventive child health care organization is obligated to appoint an internal expert on child maltreatment. The national guideline on the prevention of child maltreatment recommends that this expert is contacted each time a professional suspects child maltreatment. The interviewed experts believe this is a very important recommendation, which should always be observed.
Frequent education on the topic (of good quality)	Regularly attending a course on the prevention of child maltreatment, which also has to be of good quality.
	*“Well, what I always see as internal expert, is that if we have given a refresher course again and it's just put back on the map again, that the first two months thereafter we get phone calls more often, from people who say ‘I want to discuss this case with you, because I don’t know what to do with it.’ So they are doing it again, so in one way or another, you have to continuously bring it back to their attention, because things simply have a tendency to fade away. That works for sure.”*
Eagerness to improve oneself	When it comes to prevention of child maltreatment, professionals have to be eager to raise the bar.
***Domain: Improvement opportunities***	
**Topic**	**Description**
Fear of being wrong	Fear of being wrong or conflicting harm to the child. This fear was expected to be a very important impeding factor.
Fear of reaction	Fear of how the parent will react. This fear was expected to be a very important impeding factor.
Fear of damaging the relationship	Fear that parents will stay away after suspicions are shared with the parents. This fear was expected to be a very important impeding factor.
Fear of suffering rebuke	Fear of being rebuked by their manager, because the parents file a complaint, or because their documentation falls short. This fear was expected to be a very important impeding factor.
Fear of breaching medical confidence	Fear of breaching medical confidence and/or being punished by the board of medical examiners. This fear was expected to be an important impeding factor.
Lack of time for acting upon suspicions	When a professional signals child maltreatment, they may not always act upon it due to lack of time.
	*“It can be purely practical, like, guys, my whole day is going to be a mess if I start this conversation right now. You know what, I just didn’t see this. (…) It's not only how the other will react, it is also what all the implications are. Because then I have to call CPS and I have to do this with that person, and I have to do that, and I have to contact the school. I’m getting myself into an enormous amount of work. And maybe it will all lead to nothing. Welllll, not right now. And that is then a decision.”*
Lack of time during consultations	There is a list of actions that has to be done during each consultation, leaving little time for initiating a deep conversation.
Low self-efficacy	Low expectations of one's own ability to change the situation.
	*“If you don’t want to see it, then you won’t see it either. (…) We actually always colour our reality. Since Plato we know that already, right?”*
Difficulty referring to other disciplines	Long waiting lists, referrals that are not accepted, financial objections.
Lack of time for studying	Lack of time for studying guidelines and other literature that would aid in the prevention of child maltreatment.
Higher status	Child healthcare physicians do not feel respected by other medical specialties, leading to low self-efficacy. Some interview participants believed this negatively influences their assertiveness. Notwithstanding, they do see an improving trend concerning this issue.
	*“So I think the Calimero-effect, insecurity, not respecting their own specialty. (…) It matches their place on the medical ladder. You have to fight for that. (…) They should fight, but instead they flight or freeze.”, “We see that younger physicians are stronger, they already have some history in the hospital, during the educations, that has strengthened them. But we do see a relatively large groups that finds it difficult.(…) We need the innovators who will say, yes, it is also an opportunity to change things for the better.”*
***Items that were left out of the questionnaire***	
**Topic**	**Description**
*Home visits*	A large number of standard home visits (as determined by the organization), as well as a lower threshold to conduct a home visit on indication, is expected to enhance the prevention of child maltreatment.
*Number of standard contact moments*	The number of standard contact moments can vary a (little) bit between organizations. Most organizations don’t have a contact moment between the age of 5 and the age of 11 years. A higher number of contact moments is expected to aid the prevention of child maltreatment.
*Responsibility and compassion*	The personality traits of being more responsible and/or being more compassionate were expected to strongly impact child maltreatment prevention. Nonetheless, they were left out of the questionnaire because we do not think they can be measured reliably using self-assessment. Furthermore, they are less relevant for this study than the aforementioned topics, because it is hard to influence these areas, making them less suitable for quality improvement efforts.

***Questionnaire - English version including responses***

***Page 1 - We are glad that you decided to participate!***

**Table:** 

***Page 2***

**Table:** 

**1. What is your gender?**	
Female	97%
Male	3%

**2. For which age category do you work?**	
0–4 yr	54%
4–19 yr	21%
Both	25%
Neither (I have no direct child contact)	0.3%

**3. Which of the following educational programmes have you attended?** (Multiple responses possible)		
	Currently attending	Finished
Nursing	*(see comments)*	N=772
CHC-nursing	"	N=255 (33%)
Medicine	"	N=332
CHC-medicine	"	N=232 (70%)
Public health medicine, “Society & Health”	"	N=68 (20%)
Other finished courses (incl. non-CHC related), namely: *(textbox)*		
**Comments:**		
• The “currently attending” response option was not analysed, because it was only included to avoid response bias from CPs who currently attend a course or are nearly finished with a course. • This question actually addresses two topics: profession and specialisation. These were merged into a single question, to distract the respondent from filling in their profession. Since competition between the two types of professionals cannot be excluded, this decision may have reduced social desirability bias (i.e. “faking good”) [9].		

**4. Please fill in the following details:**	*Mean ± SD*
a. Age	46 ± 11 yr
a. Years of experience in CHC	15 ± 10 yr
a. Amount of working hours in CHC, average week	25 ± 6 h
a. Working area (Preferably specific, so we can look up population characteristics; for example:	N = 1097
a. *“(Name of city)* (Neighbourhood: *(name of neighbourhood)*)” b. “*(Name of small municipality)”* c. *“(Names of three small villages)”*	N_sufficient detail_ = 877
* in the questionnaire real names of places were used.	

***Page 3***

**Table:** 

**5. How often does your team organize an activity for parents and/or partner organizations in which you either tell them when to contact you, or in which you give them advice?**				
(E.g. a morning for teachers about signals of CM; or for parents about child rearing.)				
My team does this *(**Attention!** There are two boxes. Choose for example “1 time” in the first box, and “per year” in the second box)*				
*Dropdown menu 1*	*Dropdown menu 2*	*Transformation to 0–100 scale*		*Response distribution*
Never	Per year	0	Never	24%
1 time	Per month	16.7	≥ 1 per year	15%
2 times	Per week	33.3	≥ 1 per half year	17%
…	Per day	50	≥ 1 per 3 months	21%
9 times		66.7	≥ 1 per month	9%
10 times		83.3	≥ 1 per 2 weeks	1%
		100	≥ 1 per week	13%

**6. How well does your team know the partner organizations below?**			
*Note: For this question it's only relevant how well you know the single person that you are the most familiar with.*			
	*Not knowing who s/he is (%)*	*Knowing who s/he is (%)*	*Knowing in person (%)*
City Counsel Member	35	54	10
Police office	38	39	23
Paediatrics (nearest hospital)	16	57	27
Emergency department (nearest hospital)	83	15	2
Attention officer child maltreatment (nearest hospital)	52	24	24
Child protective services	26	54	21
**Comments:** The question is asked in a relatively factual way, which probably reduced self-overestimation. All six partner organizations were described by the interview participants as “important to know someone from, in person”, and except for the emergency department, this was also consistently described as feasible.			

**7. Which proportion of the professionals below contacts you very easily, if they suspect child maltreatment (estimation):**			
*For example, suppose 3 out of 6 GPs in your working area very easily contacts someone of your team: select the bullet in the middle.*7-point visual analogue scales ranging from “0%” to “100%”			
	*Mean (%)*	*SD (%)*	*Coefficient of variation*
**Both age categories:**			
General practitioners	26	27	1.0
Paramedics (speech therapists, physiotherapists, dieticians, …)	37	30	0.82
Child rearing experts	33	33	0.98
Social workers	44	30	0.69
Youth care workers	54	30	0.55
**0–4 yr only:**			
Midwives and maternity nurses	53	32	0.59
Preschool personnel	59	30	0.52
Special education services for pre-schoolers with disabilities	57	32	0.56
**4–19 yr only:**			
School social workers	50	32	0.64
School guidance counsellors	59	29	0.49
Teachers, age category in which CHC check-ups take place	48	29	0.61
Teachers, children of other age categories	33	27	0.82
School executives	37	32	0.85
**Comments:** All interview participants believed that it is important for a preventive child healthcare professional to personally introduce himself to relevant partners, situated in the same area. Many said that these contacts have to be renewed regularly, and remembering the partners that they should have a low threshold of contacting the child healthcare, especially when suspecting child maltreatment. Although these percentages are far below optimal care (100%), they are a lot higher than expected, considering the extreme wording (“very easily”). It is likely that this is caused by self-overestimation and inability to estimate percentages. Therefore, these results should only be appreciated relatively to each other, instead of in an absolute sense. The most worrisome finding are the three professions at the top of the table.			

***Page 4 – Impeding factors***

**Table:** 

**8. What would help you to improve your approach/efficacy when you suspect child maltreatment?**					
	*Very little (%)*	*Little (%)*	*Moderately (%)*	*Much (%)*	*Very Much (%)*
More time for each consult	5	18	38	30	9
More time for acting upon suspicions	2	6	26	52	14
More time for study and/or more knowledge/training	2	10	40	41	8
More guidance (e.g. in responding to maltreatment, or in cases of doubt)	2	10	30	48	10
Easier referring and/or shorter waiting lists	1	8	26	47	18
More certainty beforehand that I will succeed/that it will help	4	15	32	37	12
Better familiarity with partner organizations	2	8	28	50	12
More status	11	27	35	22	6

**9. Which fears do you experience when discussing maltreatment with parents and/or responding to maltreatment?**						
		Very little	Little	Moderately	Much	Very Much
Fear for:		(%)	(%)	(%)	(%)	(%)
Being wrong and/or causing harm		3	19	37	36	5
Damaging the relationship		3	16	35	40	6
An angry or violent reaction from the parent		3	25	43	27	3
Falling short in documentation		10	33	36	18	3
Violating medical confidence		11	40	33	15	1.4
Being reprimanded by my manager		30	52	15	3	0.4
Being reprimanded by the Inspectorate		19	43	26	11	1.4
Receiving a disciplinary complaint		17	39	27	14	4
***Any fear***		***0.6***	***3***	***23***	***58***	***15***
Other, namely:	N=44, including many responses similar to the following ones:					
	• *“Fear that the child will have to pay the price for it, once the parents come home”* • *“Conversational skills are insufficient”* • *“Fear of not knowing what to say”* • *“That the parents will not attend the check-ups anymore”* • *“That CPS will not help the family, after which they are returned to our care, but now with a damaged relationship”* • *“Not being ‘covered’ by colleagues from child protective services / youth care”* • *“I have not been confronted with this yet”* (* experience 0 to 3 yr) • *“Not fear, but insufficient time/possibility to plan an extra check-up”* • *“I never had to respond to maltreatment without cooperation of the parents’”*					

***Page 5 – Contact with clients (1/2)***

**Table:** 

**10. How difficult do you find it to talk with parents about the subjects below?**				
	*Easy*	*A little difficult*	*Difficult*	*Very difficult*
Alcohol or drug addiction	16	60	23	1.4
Stress, relation problems or problems at work	54	42	4	0.3
Poverty	16	60	23	1.4
Sexuality	20	51	25	4
Spousal abuse	11	50	35	4
Child maltreatment	6	45	39	9

**11. Estimate the proportion of consultations in which you act in the following ways:**7-point visual analogue scales ranging from “0%” to “100%”			
	*Mean (%)*	*SD (%)*	*Coefficient of variation*
Communicate with the parents as if you were equals	78	16	0.20
Unearthing questions, underlying questions and expectations (and answering them)	70	16	0.23
Continuously, consciously putting yourself in the parents’ shoes	76	16	0.21
Knowing the context of the client (origin, family situation, religion, social services, Intelligence, etc.)	71	17	0.23
Making your utmost effort to make the parent feel like: "What a good conversation!"	76	18	0.24
Performing the ‘parent check’ (extensively inquiring how the parents are doing, including topics as poverty, stress and alcohol consumption if you do not yet know this)	61	22	0.36
Say something nice about the child	90	14	0.16

***Page 6 – Contact with clients (2/2)***

**Table:** 

**12. Each professional behaves differently during a consult. To which degree are the following actions part of your method?**			
7-point visual analogue scales ranging from “This does not fit me at all” to “This totally fits me”.			
*(Transformed to a range from 0 to 100)*			
	*Mean*	*SD*	*Coefficient of variation*
Sharing personal mistakes with the client (if this seems meaningful for the client)	76	21	0.27
Referring a child with beginning behavioural problems to a child rearing specialist	52	27	0.52
Being able to deviate from protocols and rules (e.g. dropping a motor skills test to be able to properly listen to the parent)	83	18	0.22
Showing parents that they can tell me anything, that I am there to help them, not to judge them	87	14	0.16
Exerting pressure (e.g. by ‘threatening’ to conduct a home visit, if a parent never shows up)	27	25	0.92
**Comments:** This is an example of the “forgiving wording” technique for reducing response bias [10].			

**13. How active are you in the field of prevention of child maltreatment?**					
	*Very little (%)*	*Little (%)*	*Moderately (%)*	*Much (%)*	*Very Much (%)*
On the individual-level (case-level)	0.7	4	33	52	9
On the neighbourhood-level (e.g. influencing the policy of an entire school)	33	36	25	6	0.6
On the municipality-level	55	28	13	3	0.3
On the regional level (e.g. influencing the policy of your own organisation, or an educational institute for teachers)	67	23	7	3	1.0
On the national level	82	14	3	0.5	0.3

***Page 7 – Knowledge and skills***

**For the purpose of a new course on prevention of child maltreatment, we would like to ask that you assess yourself as honest and realistic as possible:**

**Table:** 

**14. Skills:**			
7-point visual analogue scales ranging from “low level” to “very high level”			
	*Mean*	*SD*	*Coefficient of variation*
Objective self-reflection	56	23	0.40
Being accessible to parents (ensuring that they *dare*, *want*, and *are able* to tell you anything)	74	14	0.19
Adjusting to your listener (incl. other culture, other thinking level, other reference framework)	75	15	0.20
Communicating with children, and being able to interpret the parent-child interaction	73	15	0.21
Final decision child maltreatment or not	58	17	0.29
Being assertive and showing courage	60	20	0.33
Diplomacy (tactful language, negotiating with parents, etc.)	66	17	0.26
**Comments:** The first question was only included to force the participant to think about his or her objectivity. We hoped that respondents would then try to prove themselves they responded to the first question honestly, by answering the next questions as honestly as possible.			

**15. Ready knowledge:**			
7-point visual analogue scales ranging from “low level” to “Very high level”			
	*Mean*	*SD*	*Coefficient of variation*
Contents guideline secondary prevention child maltreatment	62	19	0.31
Contents guideline child rearing support	61	20	0.33
Contents children's rights convention	44	24	0.56
Interpreting injuries (the theoretical knowledge)	55	23	0.42
Symptoms, risk factors (the theoretical knowledge)	65	19	0.30

***Page 8 - Last couple of questions…***

**Table:** 

**16. To which degree are the following statements applicable to you (in comparison to your colleagues)**					
	Very little (%)	Little (%)	Average (%)	Much (%)	Very Much (%)
My colleagues know that I’m always open to feedback	0.1	1.3	26	59	13
If something needs to be changed, I confidently bring this up to my manager	0.6	8	38	38	9
I have a sensitive personality	0.4	7	41	41	11
If a parent maltreats his/her child, I find it difficult to not judge the parent at all	4	30	53	12	1.0
I have a lot of practical experience in signalling and responding to child maltreatment	12	32	41	13	2

**17. How long ago were you a participant of a course in the following areas, and how competent did you feel 3 months later?**										
Four courses with two dropdown menus each										

*Dropdown menu 1: How long ago*			*Dropdown menu 2: Competence after 3 months*							
0–3 months ago			I did not feel competent							
4–6 months ago			I felt somewhat competent							
7–12 months ago			I felt fairly competent							
13–24 months ago			I felt very competent							
>24 months ago										
Never										

*Course*	*Not competent*			*Somewhat comp.*			*Fairly comp.*		*Very competent*	
Discussing sensitive topics	3			30			55		12	
Communicating with children	7			18			53		22	
Signalling child maltreatment	6			41			47		5	
Responding to child maltreatment	10			40			45		6	
	*Never*	*0–3 mo.*			*4–6 mo.*	*7–12 mo.*		*13–24 mo.*		*>24 mo.*
Discussing sensitive topics	*20*	*12*			*15*	*16*		*24*		*12*
Communicating with children	*12*	*5*			*6*	*6*		*26*		*44*
Signalling child maltreatment	*15*	*14*			*23*	*26*		*20*		*2*
Responding to child maltreatment	*13*	*14*			*21*	*24*		*21*		*6*

**18. If you think about the past three months, how often have you (approximately):**					
A. Been in touch with **Child Protective Services** (incl. conversations in which did not file a report of maltreatment)?					
A. Been in touch with the **designated child maltreatment expert of your own organisation**?					
*Dropdown menu 1:*	*Dropdown menu 2:*	*Transformation to a 0–100 scale*			
0	Per day	Score	Contacts within past 3 mo.	*A (%)*	*B (%)*
1	Per week	0	0	42	56
…	Per month	25	1–2	27	26
9	In total (= per 3 months)	50	3–5	24	14
10		75	6–12	4	2
		100	> 12	2	1.2

**19. Do you want to make an important comment or remark?**
(Textbox)

**Figure:**
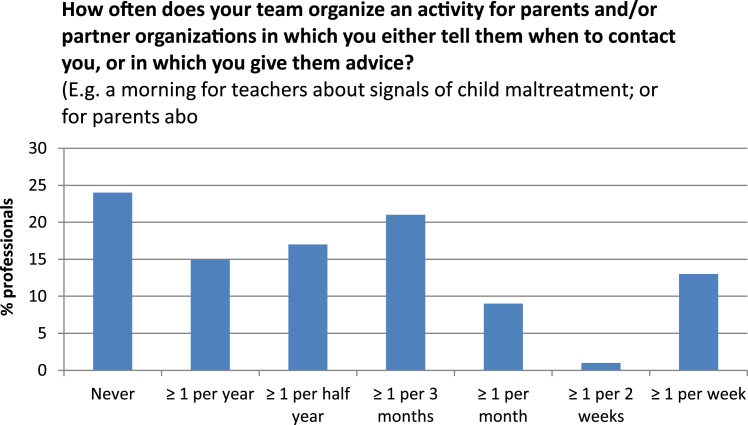

**Figure:**
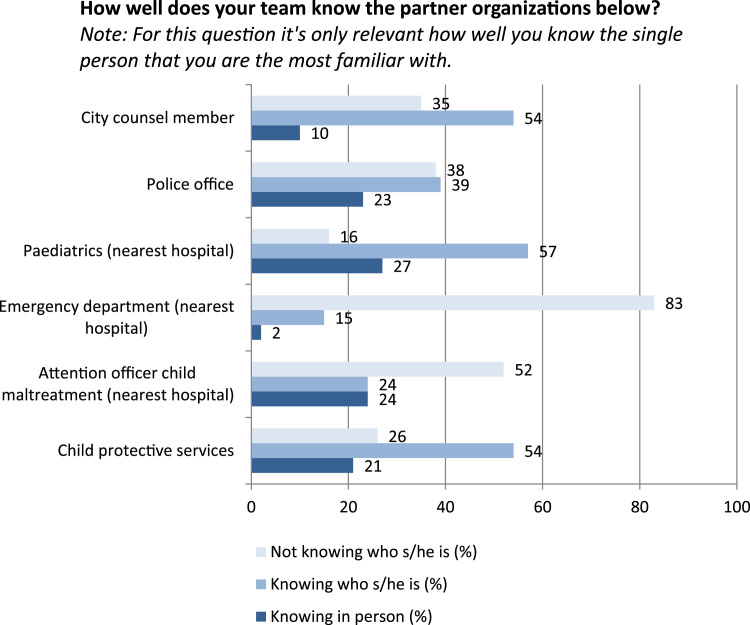

## References

[1] Visscher S., van Stel H. (2017). Variation in prevention of child maltreatment by Dutch child healthcare professionals. Child Abus. Negl..

[2] Teddlie C., Yu F. (2007). Mixed methods sampling: a typology with examples. J Mix Methods Res..

[3] M.M. Wagenaar-Fischer, N. Heerdink-Oberhuijsen, M. Kamphuis, J. de Wilde, Preventive child health care guideline secondary prevention of child maltreatment (in Dutch) Delft: Netherlands Organisation for Applied Scientific Research (TNO), 2010.

[4] Konijnendijk A.A.J., Boere-Boonekamp M.M., Haasnoot-Smallegange R.M.E., Need A. (2014). A qualitative exploration of factors that facilitate and impede adherence to child abuse prevention guidelines in Dutch preventive child health care. J. Eval. Clin. Pract..

[5] DiCicco-Bloom B., Crabtree B.F. (2006). The qualitative research interview. Med. Educ..

[6] Weston C., Gandell T., Beauchamp J., McAlpine L., Wiseman C., Beauchamp C. (2001). Analyzing interview data: the development and evolution of a coding system. Qual. Sociol..

[7] Braun V., Clarke V. (2006). Using thematic analysis in psychology. Qual. Res. Psychol..

[8] QSR International, *NVivo 10 for Windows,* 2012.

[9] Choi B.C.K., Pak A.W.P. (2005). A catalog of biases in questionnaires. Prev. Chronic Dis..

[10] Nederhof A.J. (1985). Methods of coping with social desirability bias. Eur. J. Soc. Psychol..

