# Supplementary material for: Practice variation amongst preventive child healthcare professionals in the prevention of child maltreatment in the Netherlands: Qualitative and quantitative data
Source: Data Brief. 2017 Oct 2;15:665–86. doi: 10.1016/j.dib.2017.09.061 (PMC5671414; doi:10.1016/j.dib.2017.09.061)
Supplement: Supplementary file 1 — Supplementary material [file mmc1.docx]

Conflict of Interest statement for
*“Practice variation amongst preventive child healthcare professionals in the prevention of child maltreatment in the Netherlands: qualitative and quantitative data”*

This research was not funded but performed as part of a Master’s thesis in Medicine. None of the authors have a financial or personal interest in the subject of this paper.

With regards,

Henk van Stel, PhD

assistant professor of health services research | Department of Healthcare Innovation and Evaluation

Julius Center for Health Sciences and Primary Care | University Medical Center Utrecht
